# A Glutathione Transferase from *Agrobacterium tumefaciens* Reveals a Novel Class of Bacterial GST Superfamily

**DOI:** 10.1371/journal.pone.0034263

**Published:** 2012-04-04

**Authors:** Katholiki Skopelitou, Prathusha Dhavala, Anastassios C. Papageorgiou, Nikolaos E. Labrou

**Affiliations:** 1 Laboratory of Enzyme Technology, Department of Agricultural Biotechnology, Agricultural University of Athens, Athens, Greece; 2 Turku Centre for Biotechnology, University of Turku, Åbo Akademi University, BioCity, Turku, Finland; Massachusetts Institute of Technology, United States of America

## Abstract

In the present work, we report a novel class of glutathione transferases (GSTs) originated from the pathogenic soil bacterium *Agrobacterium tumefaciens* C58, with structural and catalytic properties not observed previously in prokaryotic and eukaryotic GST isoenzymes. A GST-like sequence from *A. tumefaciens* C58 (*Atu*3701) with low similarity to other characterized GST family of enzymes was identified. Phylogenetic analysis showed that it belongs to a distinct GST class not previously described and restricted only in soil bacteria, called the Eta class (H). This enzyme (designated as *Atu*GSTH1-1) was cloned and expressed in *E. coli* and its structural and catalytic properties were investigated. Functional analysis showed that *Atu*GSTH1-1 exhibits significant transferase activity against the common substrates aryl halides, as well as very high peroxidase activity towards organic hydroperoxides. The crystal structure of *Atu*GSTH1-1 was determined at 1.4 Å resolution in complex with *S*-(p-nitrobenzyl)-glutathione (Nb-GSH). Although *Atu*GSTH1-1 adopts the canonical GST fold, sequence and structural characteristics distinct from previously characterized GSTs were identified. The absence of the classic catalytic essential residues (Tyr, Ser, Cys) distinguishes *Atu*GSTH1-1 from all other cytosolic GSTs of known structure and function. Site-directed mutagenesis showed that instead of the classic catalytic residues, an Arg residue (Arg34), an electron-sharing network, and a bridge of a network of water molecules may form the basis of the catalytic mechanism. Comparative sequence analysis, structural information, and site-directed mutagenesis in combination with kinetic analysis showed that Phe22, Ser25, and Arg187 are additional important residues for the enzyme's catalytic efficiency and specificity.

## Introduction

Glutathione transferases (GSTs; EC 2.5.1.18) are phase II detoxification enzymes that metabolize a wide range of hydrophobic toxic compounds by catalyzing the conjugation of glutathione (GSH) to the hydrophilic centre of the toxic substances [1]–[4]. GSTs are known as promiscuous enzymes capable of catalyzing the conjugation of GSH with a broad range of electrophilic substrates [5]–[7]. Several members of the GST family are selectively induced by biotic and abiotic stress treatments and play important roles in the regulation of redox homeostasis as well as in endogenous metabolism [3], [4]. GSTs can also bind hydrophobic compounds that are not their substrates [6]. This non-substrate binding (termed ‘ligandin’ function) is possibly associated with the sequestration, storage, and transportation of drugs, hormones, and other metabolites [6]. GSTs, therefore, are able to participate in various unrelated biological processes and may be considered as ‘moonlighting’ proteins [5].

GSTs form a highly diverse protein family and, therefore, have been subdivided into a number of subfamilies associated with different functionalities and enzymatic properties [8]–[12]. GSTs are divided into at least four major families of proteins, namely cytosolic GSTs, mitochondrial GSTs, microsomal GSTs, and bacterial fosfomycin-resistance proteins [5], [7], [8]. GSTs that are grouped into different classes usually have different general substrate profiles, while members of the same class have fewer differences in substrate recognition [2], [7]. All cytosolic GSTs have the same protein folding, which comprises two domains. The N-terminal domain (domain I) adopts α/β topology and provides the GSH-binding site (G-site). The C-terminal domain (domain II) is an all-α-helical structure and provides the structural element for recognition of a broad range of hydrophobic co-substrate (H-site). The H-site lies adjacent to the G-site and defines the substrate specificity of the enzyme [7]–[12].

Like eukaryotic organisms, bacteria are characterized by multiple GST genes of widely divergent sequences and unknown biological function [8]. In bacteria, four different classes of GSTs have been identified: beta, chi, theta and zeta. Most of the bacterial GSTs identified to date belong to the bacterial-specific beta class and the crystal structures of several representatives of this class have been determined and characterized, such as *Proteus mirabilis* GST (*Pm*GST) [13] and *Ochrobactrum anthropi* GST (*Oa*GST) [14].

*Agrobacterium tumefaciens* is a ubiquitous soil borne pathogen that is responsible for crown gall, the plant disease that causes large tumor-like growth in over 90 families of plants and results in major agronomical losses [15]. We have recently reported the identification and functional analysis of the GST family of enzymes from *A. tumefaciens* C58 [16]. In the present study, we report the kinetic characterization and crystal structure determination of *Atu*3701 protein from *A. tumefaciens*. Sequence and structural analysis indicate that *Atu*3701 defines a novel GST class distinct from other previously characterized GSTs.

## Results and Discussion

### Identification and bioinformatics analysis of a new class of GSTs

*In silico* homology searches of *Agrobacterium tumefaciens* strain C58 genomic sequence revealed the presence of several sequences corresponding to putative GST homologues [16]. A putative sequence with NCBI accession number AAK89703 (ORF name *Atu*3701, *Atu*GST [16]) which shares low sequence homology, and therefore significant evolutionary distance, to other prokaryotic and eukaryotic GST classes was identified and selected for further study. *Atu*GST4 contains an open reading frame of 693 bp, coding for a polypeptide of 230 amino acid residues with a predicted molecular mass of 26,140 Da (residues 1–230) and a theoretical pI of 6.33. The gene is located in a linear chromosome of *A. tumefaciens*, between 779,833–780,525 bp [17].

BLAST analysis revealed that *Atu*GST4 has the highest identity (∼64–68%) with unclassified GSTs from proteobacteria species (e.g. *Stigmatella*, *Mesorhizobium*, *Sinorhizobium*, *Bradyrhizobium*). Interestingly, several close homologs of *Atu*GST4 were found in a set of environmental sequences determined recently by the environmental (marine metagenome) sequencing project carried out by the Whole Genome Shotgun (WGS) sequencing project (www.ncbi.nlm.nih.gov/projects/WGS/WGSprojectlist.cgi). This sequence, therefore, is likely to belong to a larger family. The size of this family is expected to increase as the existing sequence databases expand.

**Figure 1:**
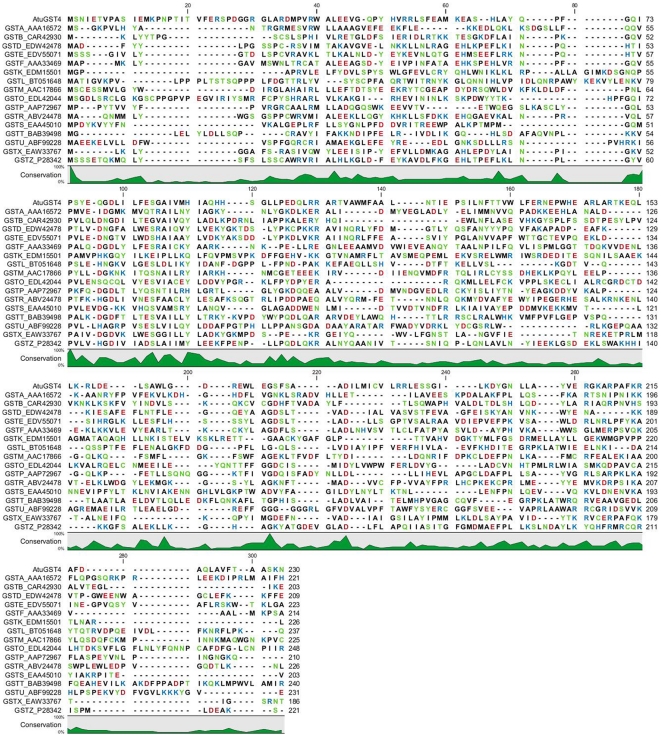
Multiple sequence alignment of *Atu*GST4 with representative GST sequences. Alpha, (GSTA, AAA16572); beta, (GSTB, CAR42930); delta, (GSTD, EDW42478); epsilon, (GSTE, EDV55071); phi, (GSTF, AAA33469); kappa, (GSTK, EDM15501); lambda, (GSTL, BT051648); mu, (GSTM, AAC17866); omega, (GSTO, EDL42044); pi, (GSTP, AAP72967); ro, (GSTR, ABV24478); sigma, (GSTS, EAA45010); theta, (GSTT, BAB39498); tau, (GSTU, ABF99228), chi, (GSTX, EAW33767); and zeta, (GSTZ, P28342). NCBI accession numbers are in parentheses. The degree of conservation is shown below the alignments in green. Amino acids are colored according to polarity or charge (red for negative charged, blue for positive charged, black for neutral and green for uncharged polar amino acids).

GSTs that share greater than 40% sequence identity are generally included in the same class, and those that possess less than 20–30% sequence identity are assigned to separate classes [5], [8], [18]. As shown in Figure 1 and Table S1, *Atu*GST4 exhibits only 17.2 to 26.1% sequence identity with representatives of all the available different GST classes, which supports the grouping of this enzyme into a new class. The *Atu*GST4 shows the highest identity with the bacterial Chi (26.1%) and plant Phi (24.2%) class enzymes.

**Figure 2:**
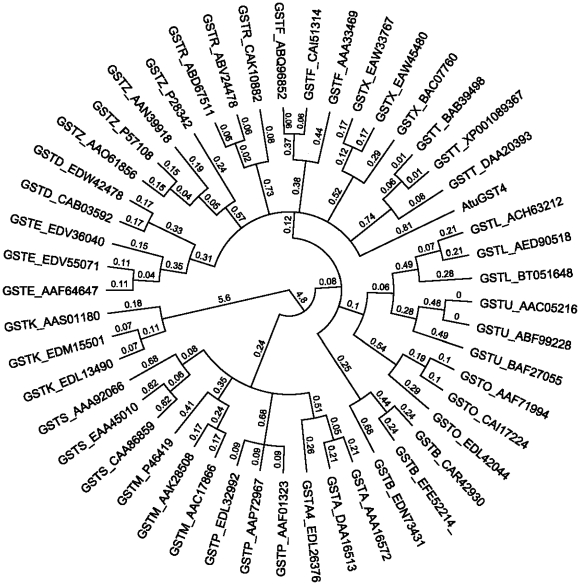
Hypothetical evolutionary history of *Atu*GST4. Phylogeny tree was constructed using representative members from all known GST classes: GSTA, (AAA16572, DAA16513 EDL26376); GSTB, (CAR42930 EFE52214 EDN73431); GSTD, (EDS36584 CAB03592 EDW42478); GSTE, (EDV55071 AAF64647 EDV36040); GSTF, (ABQ96852 CAI51314 AAA33469), GSTK, (EDL13490 EDM15501 AAS01180); GSTL, (BT051648 AED90518 ACH63212); GSTM, (AAC17866 AAK28508 P46419); GSTO, (AAF71994 CAI17224 EDL42044); GSTP, (AAP72967 EDL32992 AAF01323); GSTR, (CAK10882 ABV24478 ABD67511); GSTS, (EAA45010 CAA86859 AAA92066); GSTTtheta, (XP001089367 BAB39498 DAA20393); GSTU, (ABF99228 AAC05216 BAF27055), GSTX, (EAW45480 EAW33767 BAC07760); and GSTZ, (P57108 AAO61856 AAN39918 P28342) and the AtuGST4 from *A. tumefaciens* C58. NCBI accession numbers are in parentheses.

In order to examine the genetic relationship between this enzyme and GSTs from all known classes, a phylogenetic analysis was created (Figure 2). The results showed that the *Atu*GST4 sequence is clearly separate from all GST classes presented in the phylogenetic tree even from those representing bacterial-specific classes (e.g. beta, chi) [12], [19]. *Atu*GST4 branch extends separately from the clades of GSTB and GSTX and appears to be more ancient than them. All the above evidences point to the conclusion that *Atu*GST4 belongs to a new GST class, distinct from previously characterized GSTs. According to the available GSTs nomenclature and classification system we propose that *Atu*GST4 belongs to the Eta class (H) and may be designated as *Atu*GSTH1-1.

### Purification and kinetic analysis

In order to characterize the *Atu*GSTH1-1 protein, the full-length sequence was cloned, expressed in *E. coli* BL21 (DE3) cells, and purified. The enzyme did not bind adequately to the classical affinity adsorbents (GSH-Sepharose or hexyl-GSH-Sepharose) that are widely used for the purification of recombinant as well as native GSTs. This indicates differences in the G-site topology of *Atu*GSTH1-1 compared to the majority of other GST classes that are efficiently purified using GSH-based affinity adsorbents. *Atu*GSTH1-1 was purified (>98% purity) in a single-step procedure by metal-chelate affinity chromatography on Ni-NTA affinity adsorbent.

Steady-state kinetic analysis using CDNB and GSH was carried out and the k_cat_, and K_m_ parameters were determined (Table 1). The K_m_ values for GSH and CDNB were determined as 0.29 mM and 1.5 mM, respectively. Initial screening has shown that *Atu*GSTH1-1 exhibits high GSH-dependent peroxidase activity (GPOX) towards organic hydroperoxides such as cumene hydroperoxide and tert-butyl hydroperoxide [16]. Organic hydroperoxides can be formed both nonenzymatically by reaction of free radicals with polyunsaturated fatty acids and enzymatically by lipoxygenase- or cyclooxygenase-catalyzed oxidation of linoleic acid and arachidonic acid. *Atu*GSTH1-1 exhibits very high peroxidase activity (specific activity with cumene hydroperoxide 23.6 U/mg). With cumene hydroperoxide and tert-butyl peroxide as electrophile substrates, *Atu*GSTH1-1 exhibits high catalytic efficiency (k_cat_/K_m_) (Table 1), suggesting that hydroperoxides may be the ‘natural’ substrates for *Atu*GSTH1-1.

**Table 1:** Steady-state kinetic analysis of *Atu*GSTH1-1.

Substrate	Κ_m_ (mM)	k_cat_ (min^−1^)^a^	k_cat_/Κ_m_ (mM^−1^·min^−1^)
GSH	0.3±0.03	31.9±0.05	21.4
CDNB	1.5±0.09		
GSH	0.9±0.07	339.4±6.05	123.9
CuOOH	2.7±0.21		
GSH	1.1±0.05	149.7±5.21	95.3
t-BuOOH	1.6±0.07		
GSH	1.7±0.1	2.4±0.05	0.6
HEDS	4.1±0.12		

a k_cat_ values were calculated for the substrates CDNB, CuOOH, and HEDS.

*Atu*GSTH1-1 exhibited significant thioltransferase activity using the 2-hydroxyethyl disulfide (HED) as a substrate. The k_cat_ and K_m_ values for HED were determined as 2.4 min^−1^ and 4.1 mM, respectively (Table 1). In cases of oxidative stress, when there is a lack of GSH, some protein thiols are S-thiolated making protein-thiol disulfides [20]. This modification affects the activity of the proteins or enzymes, suggesting that *Atu*GSTH1-1 may play an important regulatory role in stress defence mechanism [21].

As shown in Table 1, the K_m_ values for GSH are dependent on the electrophilic substrate used. For example, the K_m_^GSH^ varies between 0.3 to 1.7 mM. Probably, this is the result of the rapid equilibrium random sequential bi-bi mechanism with intrasubunit modulation between the GSH binding site and electrophile binding site that is operated by GSTs [9], [10].

### Structural characterization of *Atu*GSTH1-1

#### Quality of the structure

The crystal structure of *Atu*GSTH1-1 was determined to 1.4 Å resolution with *R*_work_ and *R*_free_ of 17.2% and 18.6%, respectively (Table 2). The final refined structure contains 213 residues, 313 water molecules, 1 *S*-(p-nitrobenzyl)-glutathione (Nb-GSH) molecule, and 1 phosphate ion. The first 13 and the last 4 residues are flexible and were not modeled in the structure. Lys14 and Trp141 lack side-chains owing to their high flexibility. Ten residues were modeled in alternative conformations. The structure exhibits good stereochemistry with root mean square deviation (r.m.s.d) in bond lengths and bond angles of 0.008 Å and 1.15°, respectively. The (phi, psi) plot shows 92.5% of the non-Gly and non-Pro residues in the most favored regions and no residues in disallowed regions. One residue (Glu85) is found in the generously allowed region, possibly as a result of its interaction with Nb-GSH. The coordinate error as deduced by the diffraction precision indicator is 0.06 Å.

**Table 2:** Data collection and refinement statistics.

***Data collection***	
Space group	*C*222_1_
Cell dimensions (Å)	49.4×96.0×88.4
Number of molecules	1
Resolution range (Å)	20.0-1.40 (1.5-1.4)#
Number of measured reflections	247406 (45974)
Unique reflections	41008 (7614)
Completeness (%)	99.4 (99.9)
Mosaicity (°)	0.2
<I/σ(I)>	19.9 (4.1)
*R_merge_* (%)	5.6 (49.7)
*R*_meas_ (%)&	6.2 (54.2)
Wilson B-factor (Å^2^)	20.8
***Refinement***	
Reflections (working/test)	41008 (38919/2089)
*R*_work_/*R*_free_ (%)	17.2/18.6
Number of protein atoms	1757
Number of waters	313
*RMS deviation from ideal geometry*	
Bond lengths (Å)	0.008
Bond angles (°)	1.15
*Ramachandran plot*	
Residues in most favoured regions (%)	92.5
Residues in additional allowed regions (%)	7.0
Residues in generously allowed regions (%)	0.5
*Average B factors (Å^2^)*	
Main chain/Side chain	14.9/19.8
Waters	31.6
*S*-(p-nitrobenzyl)-glutathione	16.2

# Numbers in parenthesis correspond to the highest resolution shell.

& Redundancy-independent R-value [54].

#### Description of the structure

**Figure 3:**
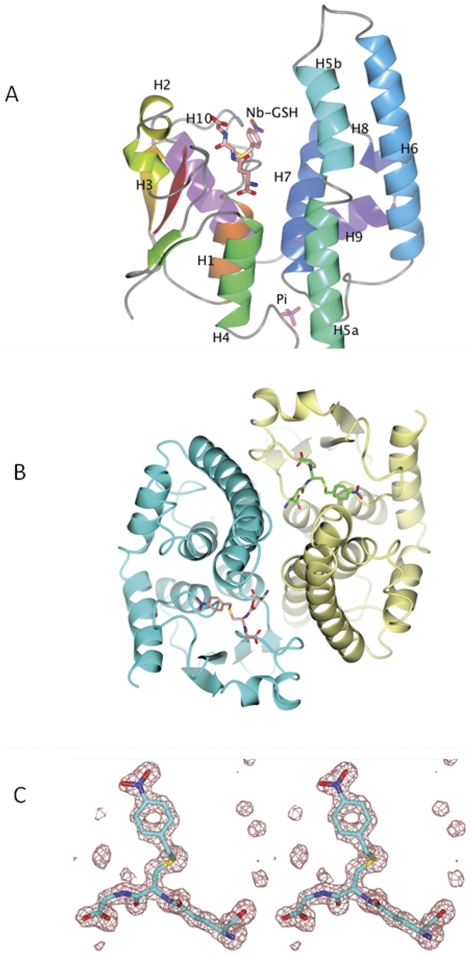
Crystal structure of *Atu*GSTH1-1. **A.** Ribbon diagram of *Atu*GSTH1-1 monomer. Assignment of secondary structure elements was carried out by DSSP [55]. The bound Nb-GSH and phosphate ion are shown as sticks coloured according to atom type. **B.** Ribbon diagram of the dimeric *Atu*GSTH1-1 structure. The 2-fold axis is perpendicular to the plane of the page. Subunit A is colored in cyan and subunit B is in lemon yellow. The inhibitor Nb-GSH is represented with sticks and coloured according to atom type. **C.** Stereo view of the mFo-DFc electron density omit map for the bound Nb-GSH contoured at 3σ. The figures were produced using the CCP4 molecular graphics program [56].

The structure of *Atu*GSTH1-1 exhibits the characteristic overall fold of GSTs that comprises an N-terminal thioredoxin-like domain and a C-terminal all α-helical domain (Figure 3) [1], [5], [9], [10]. In total, ten α-helices (H1–H10), 2 3_10_-helices and 4 β-strands (β1–β4) were located in the structure. The N-terminal domain comprises two large α-helices (H1, residues 35–45; H4, residues 85–95), two short α-helices (H2, residues 56–59; H3, residues 62–65) and a four-stranded mixed β-sheet with a left-handed twist formed by strands β2 (residues 50–54), β1 (residues 18–22), β3 (residues 75–78) and β4 (residues 81–84). Pro73 at the beginning of β3 adopts a cis-configuration and creates a characteristic turn essential for GSH binding. A 10-residue linker region (residues 96–105) that adopts an extended structure connects the N- terminal domain with the larger C-terminal domain. The latter (residues 105 to 224) has an all-α structure with the α-helices arranged in a right-handed spiral. α-Helix H5 exhibits a sharp kink at its centre (Thr121) that splits it into two smaller helices, namely H5a (residues 105–120) and H5b (residues 122–135). α-Helix H5a is straight and oriented nearly parallel to α-helix H4, while α-helix H5b has a bent appearance and projects over the active site located in the N-terminal domain. The C-terminal end of H7 takes a 3_10_-helix conformation (residues 185–190). Helices H8 (residues 197–207) and H9 (194–197) correspond closely to similar regions in most of the other GST classes. H10 (residues 203–212) folds back over the top of the N-terminal domain and against helix H1.

In the C-terminal domain, *Atu*GSTH1-1 possesses two local structural motifs, an N-capping box and a hydrophobic staple motif at the beginning of α-helix H6 in the hydrophobic core of the molecule, similar to other cytosolic GSTs [22], [23]. Both motifs are located between amino acids 172–177 (Phe-Ser-Ala-Ala-Asp-Ile). The N-capping box (Ser-Ala-Ala-Asp) consists of a reciprocal hydrogen bonding interaction of Ser173 with Asp176, whereas the hydrophobic staple motif consists of a hydrophobic interaction between Phe172 and Ile177. In mammalian GSTs and in beta class bacterial *Pm*GST these structural elements are critical for protein folding, stability, and catalytic function [8], [22], [23].

#### Structural comparison with other proteins

A Dali search [24] showed an r.m.s.d with other GSTs between 2.3–3.5 Å and a 20–25% sequence identity. The GST-like protein YfcG (PDB id 3gx0) [25] an *E. coli* GST homologue with disulfide-bond reductase activity, was identified as the closest structural neighbor of *Atu*GSTH1-1 (Z = 22.9, r.m.s.d = 1.9 Å, 22% sequence identity). The second structure in the Dali list was that of *Rhodobacter sphaeroides* GST (PDB id 3lsz; Z = 21.7, r.m.s.d = 2.5 Å, 28% sequence identity).

#### Subunit-subunit interactions

The structure of *Atu*GSTH1-1 contains one molecule in the asymmetric unit. The functional dimer found in GSTs was generated by the symmetry operator -x, y, -z+½ of the *C*222_1_ space group (Figure 3B). The interface involves 49 residues from each monomer and the buried surface area is ∼1645 Å^2^ from each monomer (about 15% of the total solvent accessible area of each monomer), which is within the values found in most other GST families [1], [7], [9], [10]. The main regions involved in subunit interactions are residues 65–72 (part of helix H3), 80–85 (strand β4), 86–96 (helix H4), 105–128 (part of helix H5), 139–143 and 154–162 from helix H6. Close inspection shows that the formation of the dimer follows the ‘lock-and-key’ mode that is also found in the phi, alpha, mu and pi classes of GSTs [9], [26]. The “lock-and-key” motif plays important functional and structural roles and is generally considered important for dimerization. The “key” is an aromatic residue in one subunit and the “lock” is a cluster of hydrophobic residues from the other interacting subunit. Indeed, the side chain of Phe70 acts as the ‘key’ that locks into a hydrophobic pocket consisting of Ile122', Leu160', Leu170', Met181', Leu200', and Trp114' from the second subunit. Six hydrogen bonds (three from each subunit) with distances from 2.5 to 3.5 Å contribute further to the stability of the interface: Arg156 NH1-Phe70 O 3.4 Å; Thr121 OG1-Glu85 OE2 2.6 Å; Arg148 NH2-Glu139 OE1 3.0 Å; Arg148 NH1-Glu139 OE2 2.8 Å. Glu85, in particular, is involved in Nb-GSH binding through its OE1 atom whereas its OE2 atom makes a hydrogen bond with Thr121 OG1 (distance 2.6 Å) from subunit B at the subunit interface. This interaction might also induce the kink of helix H5.

#### GSH Binding Site (G-site)

A molecule of Nb-GSH was found bound in the active site of *Atu*GSTH1-1 (Figure 3C). The glutathione portion of Nb-GSH is located in a region formed by the beginning of helices H1 and H4 and part of the β-turn between H3 and β3. The γ-Glu portion makes hydrogen bonds through the oxygen atoms O11 and O12 with Glu85 and Ser86. In addition, O11 makes two indirect contacts with main chain O of Pro74 and side-chain NE2 of Gln68. The N1 atom interacts with the side-chain atoms of Glu84 and also with two residues Thr121 OG1 and Asn120 NE2 from the symmetry-related subunit that forms the functional dimer. The conserved SNAIL/TRAIL motif in the N-terminal domain that is present in most GST classes and contributes polar functional groups to the GSH binding site is absent in *Atu*GSTH1-1 [27], [28]. However, a putative SNAIL/TRAIL-like motif (SGAIV) was found at amino acid positions 86–90 (Figure 1). The hydroxyl group of Ser86 makes a hydrogen bond with the γ-Glu portion of GSH. The other residues of the motif are not directly involved in GSH binding.

#### Electrophilic Binding Site (H-site)

**Figure 4:**
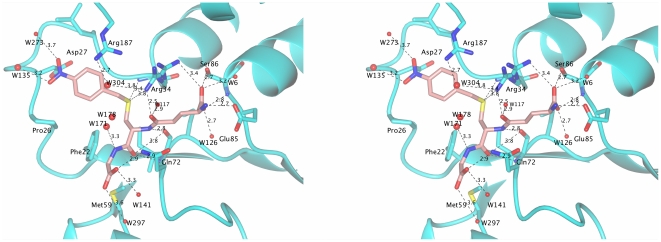
Close-up stereo view of the active site. Hydrogen-bonds (<4.0 Å) between Nb-GSH and the enzyme are shown as dashed lines. W304 and W117 from the proposed electron-sharing network are depicted. The orientation of Nb-GSH is the same as in Figure 3C. The figure was produced using the CCP4 molecular graphics program [56].

The H-site in GSTs is characterized by low conservation that reflects its role in substrate specificity. In contrast to other GSTs where the H-site involves C-terminal domain residues, interactions in *Atu*GSTH1-1 are mainly provided by Arg187 and the long turn between strand β1 and helix H1 (residues 25–33) (Figure 4). Compared to Nb-GSH binding in tau class *Gm*GSTU4-4 [9] the orientation is different with the *Atu*GSTH1-1 4-nitrobezyl group more buried than the *Gm*GSTU4-4/4-nitrobenzyl group which points towards the bulk solvent. This might be caused by the presence of bulky Trp163 in *Gm*GSTU4-4 whereas in *Atu*GSTH1-1 the structural equivalent position of Trp163 is occupied by Arg187. Further comparison shows that several hydrophobic residues from the C-terminal helix in other GSTs are absent in *Atu*GSTH1-1 as a result of the different position of the C-terminal helix (H10) in *Atu*GSTH1-1 away from the active site. In the case of alpha GSTA1-1 [29], tau *Gm*GSTU4-4 [9], and pi class GSTP1-1 [30], the C-terminal helix is longer and acts as a lid over the substrate binding site, thus creating a more restricted binding site entrance. The absence of such a feature in *Atu*GSTH1-1 may explain the ability of this enzyme to accommodate a diverse range of substrates at the H-site [16]. Salt bridges between helix H1 residues Arg34, Glu43, and Glu44 with helix H10 Arg214 and Arg209 may contribute to the stabilization of H10 position away from the active site.

### Catalytic mechanism and site-directed mutagenesis

It is widely accepted that GSTs achieve catalysis mainly through the involvement of an active site residue that interacts with and activates the sulfhydryl group of GSH to generate the catalytically active thiolate anion [1], [9]–[11], [31], [32]. This residue in the alpha, mu, pi, sigma classes is a Tyr. In the delta, epsilon, theta, tau and zeta GSTs, the active site residue is a Ser. In omega, beta and lambda is instead a catalytically essential Cys, which is involved in forming a mixed disulfide with GSH. Analysis of the structure of *Atu*GSTH1-1 showed the absence of any functional side-chain (Ser, Tyr, Cys) in hydrogen bond distance with the cysteinyl moiety of the bound Nb-GSH (Figure 4). This observation distinguishes *Atu*GSTH1-1 from all other cytosolic GSTs of known structure and function. However, structural analysis indicated that the side-chains of Phe22, Ser25, Arg34 and Arg187 are oriented towards the ligand binding-site and may be important in substrate binding and/or catalysis. Phe22 and Ser25 are located at the beginning of α-helix H2 whose structural and functional role has been established in numerous publications [9], [10], [31]. The guanidium groups of Arg187 and Arg34 are adjacent to the sulfur atom of Nb-GSH.

To investigate the role of Phe22, Ser25, Arg34 and Arg187, these residues were mutated to Ala and the mutant enzymes (Phe22Ala, Ser25Ala, Arg34Ala and Arg187Ala) were expressed in *E. coli* BL21(DE3), purified as the wild-type enzyme, and subjected to kinetic analysis. The kinetic parameters k_cat_ and K_m_ toward the two model substrates CDNB and cumene hydroperoxide were determined by steady-state kinetic analysis, and the results are listed in Table 3. The results showed that in the case of CDNB/GSH system the mutants Phe22Ala and Ser25Ala exhibit moderate differences in K_m_ values for CDNB, compared to the wild-type enzyme, indicating that the mutations do not change appreciably the affinity of the H-site for the CDNB. Small differences were also observed for the k_cat_ values. It is noteworthy that mutant Ser25Ala and Phe22Ala showed decreased K_m_ values for GSH, suggesting that these residues are involved in GSH binding in the G-site. The mutant enzyme Arg187Ala exhibits larger reduction in catalytic efficiency and shows about 3-fold lower k_cat_ value and 5-fold increase K_m_ value for CDNB, compared to the wild-type enzyme. These results suggest that Arg187 may contribute significantly either to the rate-limiting step or to the chemistry of the catalytic reaction. The mutation of Arg34 had the most detrimental effect on activity. Indeed, the Arg34Ala mutant was inactive (k_cat_ approximately 0.01 min^−1^), indicating that Arg34 may represent an important catalytic residue.

**Table 3:** Kinetic parameters of mutant enzymes for the CDNB/GSH and CuOOH/GSH reactions catalyzed by *Atu*GSTH1-1.

Enzyme	K_m_, (mM) (GSH)	K_m_, (mM) (CDNB)	k_cat_ (CDNB) (% of the wild-type)
Wild-type	0.30±0.03	1.5±0.09	100
Phe22Ala	0.085±0.002	3.2±0.39	90.5
Ser25Ala	0.14±0.01	1.9±0.18	78.2
Arg34Ala	ND^a^	ND^a^	0.01
Arg187Ala	0.34±0.08	7.1±0.76	33.5

Enzyme	K_m_, (mM) (GSH)	K_m_, (mM) (CuOOH)	k_cat_ (CuOOH) (% of the wild-type)
Wild-type	0.9±0.07	2.7±0.21	100
Phe22Ala	2.9±0.56	39.6±2.81	553.0
Ser25Ala	17.3±1.11	17.4±1.61	298.1
Arg34Ala	ND^a^	ND^a^	0.02
Arg187Ala	0.94±0.02	3.5±0.66	79.9

a ND: Non determined.

The effect of mutations using CuOOH/GSH as substrates appears to be significantly different from that seen in the CDNB/GSH system. Phe22 and Ser25 seem to play an important role in determining the K_m_ values for CuOOH since a significant increase was observed (6.4–14.7-fold). Interestingly, both mutants show also significant increase in k_cat_ values towards CuOOH. Probably, the structural integrity or flexibility of the loop where Phe22 and Ser25 are located has been altered in the mutated form of the enzyme. A plot of the crystallographic B-factors along the polypeptide chain, which can give an indication of the relative flexibility of the protein portions, indicates that this region undergo large conformational changes (data not shown). The perturbation of loop's flexibility or the loss of specific interactions may lead to structural perturbation of helix H2 with concomitant effect the alterations in K_m_ and k_cat_ values.

The mutant enzyme Arg187Ala displays moderate differences in kinetic constants, compared to the wild-type enzyme. On the other hand, the mutation Arg34 to Ala abolishes enzyme activity (k_cat_ approximately 0.02 min^−1^) using the CuOOH/GSH substrate system, providing additional evidence for the catalytic role of Arg34 (Table 3).

The effect of viscosity on the kinetic parameters was measured in order to analyze the rate-limited step of the catalytic reaction. A decrease of k_cat_ by increasing the medium viscosity should indicate that the rate-limiting step of reaction is related to the product release or to diffusion-controlled structural transitions of the protein [9], [33]–[35]. A plot of the inverse relative rate constant k_cat_^o^/k_cat_ (k_cat_^o^ is determined at viscosity η^o^) versus the relative viscosity η/η^o^ should be linear, with a slope equal to unity when the product release is limited by a strictly diffusional barrier or a slope approaching zero if the catalytic reaction chemistry is rate-limiting. The inverse relative rate constant k_cat_^o^/k_cat_ for *Atu*GSTH1-1 for the CDNB/GSH substrates system shows linear dependence on the relative viscosity with a slope 0.151±0.003 (Table 4). The observed intermediate value of the slope (0<slope<1) indicates that the rate-limiting step in the enzyme is not dependent on a diffusional barrier (i.e. product release) and other viscosity-dependent motions or conformational changes of the protein contribute to the rate-limiting step of the catalytic reaction. The effect of viscosity was also evaluated using CuOOH. The slope obtained was determined to be equal to 0.339±0.008 supporting the results obtained using CDNB as substrate. The mutants Phe22, Ser25 and Arg187 exhibit k_cat_-viscosity slopes with slight differences compared to the wild type enzyme (Table 4). This suggests that the mutations may contribute to catalysis through modulation of specific conformational changes in the enzyme without excluding the possibility of their involvement in the reaction chemistry (i.e. Arg187).

**Table 4:** The effect of viscosity on k_cat_ for the CDNB/GSH and CuOOH/GSH reactions catalyzed by *Atu*GSTH1-1 and its mutants.

Enzyme	Slope (CDNB/GSH)	Slope (CuOOH/GSH)
Wild-type	0.151±0.003	0.339±0.008
Phe22Ala	0.189±0.003	0.263±0.003
Ser25Ala	0.325±0.008	0.318±0.005
Arg187Ala	0.140±0.005	0.271±0.002

The slopes for the wild-type and the mutant enzymes were derived from the linear plot of the relative turnover number (k^o^_cat_/k_cat_) as a function of relative viscosity (η/η^o^) using glycerol as co-solvent.

Recently, a conserved electron-sharing network that assists the glutamyl γ-carboxylate of GSH to act as a catalytic base accepting the proton from the -SH thiol group of GSH, forming an ionized GSH was investigated in GSTs [36]. This electron-sharing network is created by residues that form ionic bridge interactions between the negatively-charged glutamyl carboxylate group of GSH, a positively-charged residue (primarily Arg) and a negatively-charged residue (Glu or Asp) stabilized by hydrogen- bonding networks with surrounding residues (Ser, Thr) and/or water-mediated contacts. This network has been suggested to contribute to the “base-assisted deprotonation” model postulated to be essential for the GSH ionization step of the catalytic mechanism [36]. In the *Atu*GSTH1-1/Nb-GSH complex, the conserved residues Arg34, Glu85, Ser86, Gln68 and Asn120' appear to form the proposed electron-sharing network. Based on Quantum mechanics/Molecular mechanics (QM/MM) calculations it was recently proposed [37] that the GSH activation by GSTs is accomplished by a water-assisted proton-transfer mechanism that takes into account the suggested roles of the GSH γ-glutamyl carboxylate group and the active-site water molecules. According to this mechanism, a water molecule acting as a bridge is able to transfer the proton from the GSH thiol group to the GSH γ-glutamyl carboxylate group. Dourado *et al.* have resorted to density functional theory and to potential of mean force calculations to determine the GSH activation mechanism of GSTP1-1 and GSTM1-1 isoenzymes [37]. For the GSTP1-1 enzyme, they have demonstrated that a water molecule can assist a proton transfer between the GSH cysteine thiol and the GSH glutamate alpha carboxylate groups. In the case of GSTM1-1 enzyme, two water molecules positioned between the GSH-SH and the N atom of His107, working like a bridge, are able to promote the proton transfer between these two active groups. Arg34 in *Atu*GSTH1-1 occupies two alternative positions and exhibits high crystallographic temperature factors, indicating significant flexibility. In particular, in one conformation its guanidium group interacts with the γ-glutamyl carboxylate of GSH forming an electrostatic interaction, while in the second conformation with the sulfur atom of Nb-GSH (3.4 Å), the water molecules W117 and W304, and the backbone carbonyl group of bound Nb-GSH. Arg187 interacts with the water molecule W304 and forms a π-cation interaction with the benzyl group of Nb-GSH. Hence, Arg34 and Arg187 appear to work as a bridge that connects the two water molecules 304 and 117 (Figure 4). Wat304 might be a crucial element in the catalytic mechanism. In the structure, Wat304 was found fixed by the guanidium group of Arg187 with a hydrogen bond of 2.7 Å and with Arg34 with one weak hydrogen bond (3.8 Å). The sulfur atom of Nb-GSH is 5.1 Å away from Wat304. The residues Arg187 and Arg34 could, therefore, function as a ‘clamp’ to grip Wat304 in a position to form a hydrogen bond with the sulfonate group. Based on the above analysis, in the case of *Atu*GSTH1-1, a putative bridge of a network of water molecules in the region of an electron-sharing network does exist as shown in Figure 4. Consequently, Arg34 may act as a catalytic residue for GSH activation.

In conclusion, in the present study we showed the structural and functional characterization of the *Atu*3701 protein from *A. tumefaciens*. Sequence and structural analysis indicated that *Atu*3701 defines a new GST class. Based on the available GSTs nomenclature and classification system the new class was classified as the Eta class (H) and accordingly the enzyme was named *Atu*GSTH1-1. Members of this class were found in soil bacteria and more recently in a set of environmental sequences. Thus, this structure most likely represents a larger family, whose size is expected to grow further as the existing sequence databases expand. *Atu*GSTH1-1 exhibits wide substrate specificity although analysis of the catalytic efficiency (k_cat_/K_m_) suggests that hydroperoxides may be its ‘natural’ substrates, indicating that the enzyme may play important role in counteracting oxidative stress conditions. Investigation of the crystal structure of *Atu*GSTH1-1 in complex with Nb-GSH indicated that although the enzyme adopts the canonical GST fold it lacks the classic catalytic essential residues in GSTs (e.g. Tyr, Ser, Cys). This characteristic distinguishes *Atu*GSTH1-1 from all other cytosolic GSTs of known structure and function. Site-directed mutagenesis showed that Arg34 may represent the catalytic residue. This residue together with an electron-sharing network and a bridge of water molecules are proposed to form the basis of the catalytic mechanism.

## Materials and Methods

### Materials

Reduced glutathione, 1-chloro-2,4-dinitrobenzene (CDNB), Nb-GSH and all other enzyme substrates and chemicals were obtained from Sigma-Aldrich, USA. Molecular biology reagents were purchased from Invitrogen, USA.

### Cloning, expression, and purification of *Atu*GSTH1-1 in *E. coli*

Cloning and expression of *Atu*GSTH1-1 in *E. coli* BL21(DE3) cells was carried out as described previously [16]. Purification of *Atu*GSTH1-1 was carried out as following: after expression, *E. coli* BL21(DE3) cells were harvested by centrifugation at 10,000 g for 10 min (4°C), resuspended in potassium phosphate buffer (50 mM, pH 8.0, 9 ml) containing sodium chloride (0.3 M), sonicated, and centrifuged at 10,000 g for 20 min. The supernatant was collected and was loaded to a column of Ni-NTA adsorbent (1 ml), which was previously equilibrated with potassium phosphate buffer (50 mM, pH 8.0) containing sodium chloride (0.3 M). Non-adsorbed protein was washed off with 10 ml equilibration buffer, followed by 20 ml of potassium phosphate buffer (50 mM, pH 6.2) containing sodium chloride (0.3 M) and glycerol (10%, v/v). Bound *Atu*GSTH1-1 was eluted with equilibration buffer containing imidazole in gradually increasing concentrations of 5 mM, 20 mM, 0.1 M, 0.2 M and 0.5 M (total volume of 10 ml). Collected fractions (2 ml) were assayed for GST activity and protein (Bradford assay). Fractions with *Atu*GSTH1-1 activity were pooled and dialysed overnight against appropriate buffer and was used for kinetics and structural analysis. Protein purity was judged by SDS-PAGE.

### Bioinformatic analysis

Multiple sequence alignment and phylogenetic analysis were carried out as described by Skopelitou *et al.* (2012) [16].

### Assay of enzyme activity and protein

Enzyme assays were performed according to Skopelitou et al. [16]. Observed reaction velocities were corrected for spontaneous reaction rates when necessary. All initial velocities were determined in triplicate in buffers equilibrated at constant temperature. Turnover numbers were calculated on the basis of one active site per subunit. One unit of enzyme activity is defined as the amount of enzyme that catalyses the turnover of 1 µmol of substrate per min. Specific activity is expressed in µmol · min^−1^ per mg of protein. Protein concentration was determined by the Bradford assay using bovine serum albumin (fraction V) as standard. Steady-state kinetic measurements for the wild-type enzyme were performed at 37°C in 0.1 M potassium phosphate buffer, pH 6.5, over 10-fold varied substrate concentrations. Steady-state data were fitted to the Michaelis-Menten equation by nonlinear regression analysis using the GraFit (Erithacus Software Ltd.) computer program.

### Viscosity dependence of kinetic parameters

The effect of viscosity on kinetic parameters was assayed in 0.1 M potassium phosphate buffer, pH 6.5, containing variable glycerol concentrations. Viscosity values (η) were calculated as described in Wolf *et al* [38].

### Site-Directed Mutagenesis

Site-directed mutagenesis was performed according to Ho *et al* [39]. The pairs of oligonucleotide primers used in the PCR reactions were as follows: for the Ser25Ala mutation, 5′-CGTTTTTGAACGCGCGCCCGATGGCGG-3′ and 5′- CCGCCATCGGGCGCGCGTTCAAAAACG-3′ for the Phe22Ala mutation, 5′-CGATCACCGTTGCGGAACGCTCTCC-3′ and 5′-GGAGAGCGTTCCGCAACGGTGATCG-3′, for the Arg34Ala mutation, 5′- GGTCTCGCGGCGGATATGCCG-3′ and 5′-CGGCATATCCGCCGCGAGACC-3′, for the Arg187Ala mutation, 5′- CGTCTTACGCGCGCTGGAATCG-3′ and 5′-CGATTCCAGCGCGCGTAAGACG-3′. All mutations were verified by DNA sequencing. The mutant enzymes were expressed and purified as described for the wild-type enzyme.

### Crystallization

Prior to crystallization, *Atu*GSTH1-1 was concentrated to 4.85 mg/ml in buffer Tris-HCl 15 mM, pH 7.0 and mixed with a 100 mM stock solution of *S*-(p-nitrobenzyl)-glutathione (10 mM final concentration). Crystals were grown with the hanging drop vapor diffusion method. The drops contained 2 µl of the protein solution mixed with 2 µl of a well solution (1.4 M Na/K phosphate, pH 8.3). The drops were equilibrated against 800 µl of well solution at 16°C.

### Structure determination and refinement

An initial data set to 1.4 Å resolution was collected on station X13 at EMBL-Hamburg c/o DESY from a single crystal soaked for a few seconds in crystallization solution supplemented with 20% v/v glycerol as cryoprotectant. The crystal was subsequently placed in a gaseous nitrogen stream and flash-cooled directly at 100 K. A total of 250 images were recorded on a MARCCD detector using a rotation angle of 0.5° and an exposure time of 10 seconds. Data were processed with XDS [40]. Crystals of *Atu*GSTH1-1 were found to belong to the *C*222_1_ space group with unit cell dimensions 49.4×96.0×88.4 Å. Assuming one molecule in the asymmetric unit, the Matthews coefficient [41] is 2.3 Å^3^/Da^−1^, corresponding to 46.5% solvent content. Attempts to determine the structure by molecular replacement did not produce any clear solution as judged by the low Z-scores (below 5) in PHASER [42] and the poor quality of the resultant electron density maps. Initial phases were obtained by Br-SAD from a single *Atu*GSTH1-1 crystal soaked with 1 M KBr for 45 seconds in cryoprotectant solution. The crystal was subsequently flash-cooled to 100 K in a stream of gaseous N_2_. A total of 600 diffraction images were collected (λ = 0.9 Å) to 2.01 Å resolution on the BW7A beamline at EMBL-Hamburg (c/o DESY) using a rotation angle of 0.5°, exposure time of 1 sec per image, and a MARCCD detector. Data were processed with DENZO and Scalepack [43]. The search for Br atoms was performed with SHELX [44], which identified an anomalous signal of 1.2 up to 2.4 Å resolution and located 8 Br ions. Phasing with AutoSHARP [45] resulted in a phasing power of 1.125 and an initial figure-of-merit of 0.3. Following solvent flattening and density modification, ARP/wARP [46] was able to build 202 residues in 3 chains out of 227 residues in total in the aminoacid sequence. Refinement was initially carried out with REFMAC [47] and slowly extended to 1.4 Å in small steps of 0.2 Å. At the final stages of refinement, the program PHENIX [48] was employed. No anisotropic B-factor refinement was performed as the drop in *R*_free_ was insignificant. The structure was visualized and rebuilt using COOT [49]. MOLPROBITY [50] and PROCHECK [51] were used to validate the structure. Structural superpositions were performed with SSM [52] and analysis of interfaces with PDBePISA [53].

### Protein Data Bank accession code

The final coordinates and the structure factors have been deposited with the Protein Data Bank under the accession code 2ycd.

## Supporting Information

Table S1 **Aminoacid sequence homology between** ***Atu*** **GST4 and representative GST sequences from classes:** alpha, (Q08392); beta, (P15214); delta, (B4HHD9); epsilon, (B3NMR7); phi, (P12653); kappa, (P24473); lambda, (B7FHT3); mu, (P21266); omega, (Q8K2Q2); pi, (P09211); ro, (Q0GZP3); sigma, (P46428); theta, (P30711); tau, (Q10CE7), chi, (Q8DMB4); and zeta, (P28342).
(DOC)
