# Supplementary material for: A Glutathione Transferase from Agrobacterium tumefaciens Reveals a Novel Class of Bacterial GST Superfamily
Source: PLoS One. 2012 Apr 4;7(4):e34263. doi: 10.1371/journal.pone.0034263 (PMC3319563; doi:10.1371/journal.pone.0034263)
Supplement: Table S1 — Aminoacid sequence homology between Atu GST4 and representative GST sequences from classes: alpha, (Q08392); beta, (P15214); delta, (B4HHD9); epsilon, (B3NMR7); phi, (P12653); kappa, (P24473); lambda, (B7FHT3); mu, (P21266); omega, (Q8K2Q2); pi, (P09211); ro, (Q0GZP3); sigma, (P46428); theta, (P30711); tau, (Q10CE7), chi, (Q8DMB4); and zeta, (P28342). (DOC) [file pone.0034263.s001.doc]

**Supplementary material**

**Suplementary Table 1.** Aminoacid sequence homology between *Atu*GST4 and representative GST sequences from classes: alpha, (GSTA, AAA16572); beta, (GSTB, CAR42930); delta, (GSTD, EDW42478); epsilon, (GSTE, EDV55071); phi, (GSTF, AAA33469); kappa, (GSTK, EDM15501); lambda, (GSTL, BT051648); mu, (GSTM, AAC17866); omega, (GSTO, EDL42044); pi, (GSTP, AAP72967); ro, (GSTR, ABV24478); sigma, (GSTS, EAA45010); theta, (GSTT, BAB39498); tau, (GSTU, ABF99228), chi, (GSTX, EAW33767); and zeta, (GSTZ, P28342).

| **Enzyme** | ***Atu*GSTH1-1** | **GSTA** | **GSTB** | **GSTD** | **GSTE** | **GSTF** | **GSTK** | **GSTL** | **GSTM** | **GSTO** | **GSTP** | **GSTR** | **GSTS** | **GSTT** | **GSTU** | **GSTX** | **GSTZ** |
| --- | --- | --- | --- | --- | --- | --- | --- | --- | --- | --- | --- | --- | --- | --- | --- | --- | --- |
| ***Atu*GSTH1-1** | 100 | 18.6 | 22.5 | 19.5 | 18.3 | 24.2 | 19.1 | 18.5 | 20.9 | 19.1 | 24.1 | 24.1 | 17.2 | 20.5 | 22.1 | 26.1 | 21.7 |
| **GSTA** | 18.6 | 100 | 22.3 | 22.5 | 23.7 | 20.9 | 14.6 | 17.5 | 24.6 | 19.1 | 29.5 | 16.7 | 24.6 | 22.5 | 19.1 | 22.1 | 24.4 |
| **GSTB** | 22.5 | 22.3 | 100 | 24.3 | 24.2 | 26.6 | 20.2 | 19.7 | 17.5 | 22.3 | 21.3 | 22.5 | 21.9 | 23.3 | 21.1 | 28.3 | 26.7 |
| **GSTD** | 19.5 | 22.5 | 24.3 | 100 | 38.1 | 25.8 | 14.8 | 19.9 | 15.6 | 17.8 | 18.6 | 23.8 | 18.7 | 23.7 | 23.6 | 27.8 | 26.5 |
| **GSTE** | 18.3 | 23.7 | 24.2 | 38.1 | 100 | 29.4 | 20.3 | 20.8 | 21.4 | 20.0 | 21.0 | 25.7 | 19.8 | 28.2 | 24.6 | 27.4 | 32.2 |
| **GSTF** | 24.2 | 20.9 | 26.6 | 25.8 | 29.4 | 100 | 17.8 | 19.6 | 22.5 | 19.5 | 24.0 | 26.4 | 19.7 | 23.7 | 19.7 | 31.6 | 26.5 |
| **GSTK** | 19.1 | 14.6 | 20.2 | 14.8 | 20.3 | 17.8 | 100 | 17.0 | 16.9 | 17.5 | 19.1 | 16.5 | 18.7 | 22.5 | 18.5 | 20.0 | 21.9 |
| **GSTL** | 18.5 | 17.5 | 19.7 | 19.9 | 20.8 | 19.6 | 17.0 | 100 | 18.1 | 23.8 | 17.0 | 18.5 | 17.6 | 23.5 | 22.0 | 21.4 | 23.6 |
| **GSTM** | 20.9 | 24.6 | 17.5 | 15.6 | 21.4 | 22.5 | 16.9 | 18.1 | 100 | 18.1 | 30.1 | 18.1 | 24.3 | 21.2 | 19.5 | 18.7 | 20.8 |
| **GSTO** | 19.1 | 19.1 | 22.3 | 17.8 | 20.0 | 19.5 | 17.5 | 23.8 | 18.1 | 100 | 22.1 | 23.2 | 17.6 | 20.3 | 23.2 | 19.8 | 16.3 |
| **GSTP** | 24.1 | 29.5 | 21.3 | 18.6 | 21.0 | 24.0 | 19.1 | 17.0 | 30.1 | 22.1 | 100 | 19.8 | 25.6 | 20.5 | 17.2 | 25.4 | 22.7 |
| **GSTR** | 24.1 | 16.7 | 22.5 | 23.8 | 25.7 | 26.4 | 16.5 | 18.5 | 18.1 | 23.2 | 19.8 | 100 | 20.3 | 22.2 | 22.0 | 27.2 | 29.9 |
| **GSTS** | 17.2 | 24.6 | 21.9 | 18.7 | 19.8 | 19.7 | 18.7 | 17.6 | 24.3 | 17.6 | 25.6 | 20.3 | 100 | 18.2 | 16.7 | 23.7 | 18.2 |
| **GSTT** | 20.5 | 22.5 | 23.3 | 23.7 | 28.2 | 23.7 | 22.5 | 23.5 | 21.2 | 20.3 | 20.5 | 22.2 | 18.2 | 100 | 23.6 | 24.9 | 26.0 |
| **GSTU** | 22.1 | 19.1 | 21.1 | 23.6 | 24.6 | 19.7 | 18.5 | 22.0 | 19.5 | 23.2 | 17.2 | 22.0 | 16.7 | 23.6 | 100 | 23.4 | 26.9 |
| **GSTX** | 26.1 | 22.1 | 28.3 | 27.8 | 27.4 | 31.6 | 20.0 | 21.4 | 18.7 | 19.8 | 25.4 | 27.2 | 23.7 | 24.9 | 23.4 | 100 | 29.9 |
| **GSTZ** | 21.7 | 24.4 | 26.7 | 26.5 | 32.2 | 26.5 | 21.9 | 23.6 | 20.8 | 16.3 | 22.7 | 29.9 | 18.2 | 26.0 | 26.9 | 29.9 | 100 |
